# Demographic and clinical profile of patients utilising a transitional care intervention in the Western Cape, South Africa

**DOI:** 10.4102/sajpsychiatry.v26i0.1523

**Published:** 2020-08-26

**Authors:** Henmar F. Botha, Liezl Koen, Daniel J.H. Niehaus, Yanga Vava, Karis Moxley, Ulla Botha

**Affiliations:** 1 Department of Psychiatry, Faculty of Medicine and Health Sciences, Stellenbosch University, Cape Town, South Africa

**Keywords:** deinstitutionalisation, step-up or step-down facilities, transitional care, intermediary care, community-based care, schizophrenia, psychosocial rehabilitation, South Africa

## Abstract

**Background:**

The World Health Organization’s action plan for 2020 has identified the need for service-based data to motivate for more appropriate community-based services. To date, there is no published data from step-up or step-down facilities in South Africa.

**Aim:**

To describe the demographic and clinical profile of all patients admitted to New Beginnings between 01 January 2011 and 31 December 2015.

**Setting:**

New Beginnings is an intermediary care facility focused on psychosocial rehabilitation and accommodates 40 patients in a step-up or step-down setting.

**Methods:**

In this retrospective audit, we reviewed the medical records of all patients (*N* = 730) admitted to New Beginnings between 01 January 2011 and 31 December 2015.

**Results:**

Most admissions were male (*n* = 600; 82.2%), unmarried (92.1%) and unemployed (92.7%) patients with a mean age of 28 years. Only 20.7% had completed their schooling and 37.9% were receiving a disability grant. Most patients lived in the Cape Town Metro area (89%) with their families (94.7%), and 75.6% had no children. Schizophrenia (53.7%) was the most common primary psychiatric diagnosis, and most patients were on a combination of oral and depot treatment (46.8%). Illicit substances were used by 75.9% of patients with 30% using both cannabis and methamphetamine. Most patients (74.9%) had only one admission to New Beginnings.

**Conclusions:**

These baseline data could inform improved service delivery. Further research is needed to evaluate the success of New Beginnings and highlight the need for more of these facilities in the Western Cape and across South Africa.

## Introduction

Deinstitutionalisation in South Africa started in the early 1990s but has not been accompanied by the development of community-based multidisciplinary mental healthcare.^1,2,3,4^ The reason for this is multifactorial and includes mental healthcare budget challenges, especially at community level, lack of adequate information systems, lack of development of community-based services appropriate to the severity of the mental health condition, lack of facilities providing psychosocial rehabilitation and lack of engagement with users and families.^5^ Furthermore, the decreased number of inpatient psychiatric beds has put increased pressure on available beds.^6^

Consequently, premature discharges are necessary to ensure that more severely, ill patients can be accommodated. This has resulted in high readmission rates, revolving door patterns of care and high-frequency users.^7^ These consequences have placed much emphasis on the recovery model and how it can be optimised.

Recovery in severe mental illness refers to both internal conditions (hope, healing, empowerment, connection, personal autonomy, social identity, meaning and purpose) and external conditions (implementation of human rights, a positive culture of healing and recovery-oriented services).^8^ Likewise, a multidisciplinary approach – not only involving pharmacotherapy but also psychosocial interventions and attention to environmental circumstances – can improve outcomes.^9^ Hence, the focus should be on intermediary facilities that allow acute beds to be freed up while giving patients time to stabilise, receive psychosocial interventions and adequately plan discharge and follow-up.

Transitional interventions can be divided into pre-discharge, post-discharge and bridging interventions.^10,11^ Pre-discharge interventions include psychosocial skills training, medication reconciliation and/or education, structured needs assessment and scheduling a follow-up appointment prior to discharge. Post-discharge interventions include telephonic follow-ups and home visits, efforts to ensure psychiatric follow-up, psycho-education, family education and peer support. Bridging interventions include communication of the discharge plan to the community healthcare provider, meeting with the community healthcare provider and the use of a transitional manager.^10,11^ Illness severity on discharge is likely to have a significant impact on patients’ ability to understand their treatment plan and benefit from discharge planning, which has been found to be a major predictor of readmission.^12^ Hengartner et al. emphasised that the success of these services relies on a carefully planned and coordinated transition from inpatient to outpatient care.^13^ Step-up or step-down facilities are an example of a service that bridges the gap between inpatient and outpatient care.

In high-income countries, such as Australia, facilities were established to improve recovery-focused rehabilitation services and use an integrative recovery-oriented model for people with severe mental illness.^14^ Despite some successes, there is still need for further development of community-based psychosocial rehabilitation programmes harnessing task shifting and self-help strategies.^3^

The WHO Mental Health Action Plan for 2020 emphasises the need to provide comprehensive, integrated and responsive mental health and social care services in community-based settings and to strengthen research for mental health.^15^ Studies conducted as part of the Programme for Improving Mental Healthcare (PRIME) consortium have identified the great need for research to identify optimal service utilisation indicators in South Africa and other low- to middle-income countries (LMICs).^16^

Information on current service resources (budgets, staff, facilities) and provision (admissions, outpatient visits) is extremely sparse. Of concern is that even though there are reportedly 63 community residential facilities in South Africa,^17^ very little data have emerged from these sites.

This possibly highlights insufficient investment into the development of community-based psychosocial rehabilitative services in South Africa.^3^ Community-based care can be a cost-effective strategy of achieving broader patient coverage and improved functioning, but it requires careful planning, flexible budgeting, policy commitment and appropriate resource allocation.^18,19^ Overall, the lack of data is an important limitation considering the urgent and growing need to further develop community-based mental health services.^17^

New Beginnings is an example of a well-established South African intermediary facility. The facility was launched in 2008 and is situated close to Stikland Hospital, Cape Town. The facility aims to relieve pressure on the acute psychiatric services by providing a continuum of care from acute hospitalisation to community-based rehabilitation services. New Beginnings provides an intensive psychosocial rehabilitation service to mental healthcare users requiring longer stay in a structured environment. Patients participate in an 8- to 12-week voluntary programme that includes daily walks and exercise groups; education on mental illness, medication, comorbid substance abuse and general well-being; life skills groups; daily chores for independent living; craft projects to stimulate creativity; volunteer groups; and recreational drumming.

Caregivers are engaged as part of the programme to ensure their readiness to take on the caregiver role and have the necessary knowledge and support (Internal Stikland document, personal communication, March 13, 2018).

Patients are discharged to caregivers and receive psychiatric follow-up at their nearest community health centre or day hospital. New Beginnings provides an after-care support service for discharged patients and a step-up service to previously admitted persons who require some assistance with their recovery, usually for a 2-week admission period. Step-up is often used to prevent relapse in patients who are exhibiting signs of imminent relapse, but are not ill enough to justify admission. New Beginnings has been functioning for a decade, but very little data have emerged from the facility.

The dearth of data available for South African intermediary facilities hinders healthcare planning and the improvement of community-based service delivery. As a first step towards rectifying this, there is a need to provide a systematic account of patients who have accessed care at New Beginnings. These data may highlight the key issues that need to be addressed to improve the care provided by this facility and other community-based services in South Africa.

## Methods

### Study design

We conducted a retrospective audit of patients admitted to New Beginnings between 01 January 2011 and 31 December 2015. This period was selected to allow for a post-inclusion period of 24 months, which will be reported in a follow-up study.

### Study setting

New Beginnings is situated in Bellville, Cape Town, and was launched in 2008 because of the increasing need for acute psychiatric services. It is an intermediary care facility focused on psychosocial rehabilitation and accommodates 40 patients in a step-up or step-down setting. Patients discharged from the acute inpatient wards at Stikland Hospital and needing further psychosocial rehabilitation may be referred. These patients are stabilised on psychotropic medication and referred to New Beginnings as voluntary patients. New Beginnings is regarded as a ward of Stikland Psychiatric Hospital from a management perspective and is funded by the Department of Health. All medication comes from the Stikland Hospital budget and patients go to the Stikland outpatient department on a monthly basis for review and treatment by a medical officer. The main focus of the programme is not only on stabilisation of acute episodes of serious mental illness (in comparison to acute inpatient services) but also on empowerment, skills acquisition and structuring to individual patient needs. The staff complement of New Beginnings includes a facility manager, occupational therapist, occupational therapist technician, social worker, operational manager, administrative management team consisting of two administrative clerks, a facility driver and a housekeeper, six professional nurses, seven enrolled nurses, eight nursing assistants, two cooks, four cleaners and three security guards (day and night). The mental health staff are trained professionals with experience in psychosocial rehabilitation and psychiatry. A medical officer (based at Stikland Hospital) provides care as needed and there is access to a psychiatrist if more input is required. State psychiatrists are joint appointments, two-thirds clinical and one-third academic. New Beginnings also make use of contracted home-based carers who work on rotational shifts and guide the patient programme in terms of activities of daily living (self-care, chores, meal preparation, clothing care, escorting patients to appointments and use of leisure exploration activities).

Criteria for admission to New Beginnings are summarised in Box 1.

BOX 1 General criteria for admission to New Beginnings (Internal Stikland document, personal communication, March 13, 2018).

- Mental healthcare user from the drainage area with an identified need for psychosocial rehabilitation.
- Client should be motivated to participate in the voluntary programme for a minimum of 8 weeks and a maximum of 12 weeks.
- Must be able to go on weekend leave as part of reintegrating into the community.
- Must be on psychiatric treatment and compliance on medication is emphasised.
- Must have a clear exit plan.
- General adult psychiatry age limits (18–59, extremes included).

### Study sample

We included all admissions (both male and female patients) to New Beginnings between 01 January 2011 and 31 December 2015. No exclusion criteria were identified. The total number of patients admitted during this period was 730 people.

### Data collection

Patient files were retrieved from the New Beginnings archives storing unit. Other data were extracted from the Clinicom Application Manager (a Western Cape hospital database keeping record of patient demographics, outpatient appointments and hospital admissions). All data were collated on a Microsoft Excel spreadsheet. Data extracted from the patient charts included demographic information (age, gender, residential area, language, highest level of education, marital status, number of children, employment, primary carer and disability grant) and clinical information (assertive community treatment [ACT] patient, primary and secondary diagnosis, number of psychiatric inpatient admissions and total days in hospital, number of New Beginnings admissions and total days in New Beginnings, medication, comorbid substance use and treatment for medical conditions).

Data extracted from Clinicom included all past psychiatric hospital admission dates.^17^ Diagnoses were made in the clinical setting, based on the Diagnostic and Statistical Manual of Mental Disorders IV, fourth edition, text revision (DSM-IV-TR) or the Diagnostic and Statistical Manual of Mental Disorders, fifth edition (DSM-V) diagnostic criteria.^20^ Formal questionnaires were not routinely used.

### Data analysis

Continuous variables were summarised as mean and standard deviation, while nominal variables were summarised as counts and percentages. As this was a descriptive study, no inferential statistics were employed.

### Ethical consideration

Ethical clearance was obtained from the Health Research Ethics Committee of Stellenbosch University (study number: 6965), as well as from the Head of Establishment (Stikland Hospital). A waiver of informed consent was granted for this retrospective study. All data were anonymised to ensure privacy and confidentiality of patients’ personal information, with each participant assigned a unique identifier.

The study was approved by the Health Research Ethics Committee (HREC) of Stellenbosch University (reference number: S18/04/081) as well as the Research and Ethics Committee at Stikland Hospital (WC_201805_015).

## Results

The demographic characteristics of 730 patients who were admitted to New Beginnings step-up or step-down facility from 01 January 2011 to 31 December 2015 are summarised in Table 1.

**TABLE 1:** Service-related data and demographic characteristics of patients (*N* = 730) admitted to New Beginnings step-up or step-down facility from 01 January 2011 to 31 December 2015.

Variable	*n*	%
**Gender**		
Male	600	82.2
Female	130	17.8
**Throughput per bed (2011–2015)**		
Male	18.75	-
Female	16.25	-
**Age groups**		
18–19	49	6.7
20–29	424	58.1
30–39	179	24.5
40–49	61	8.4
50–59	17	2.3
**First language**		
Afrikaans	548	75.1
Xhosa	90	12.3
English	84	12.5
Other†	8	1.1
**Highest level of education**		
Gr.1–6	69	9.5
Gr.7–9	252	34.5
Gr.10–11	146	20.0
≥ Gr.12	151	20.7
Missing data	112	15.3
**Employment**		
Unemployed	677	92.7
Casual employment	34	4.7
Formal employment	19	2.6
**Disability grant**		
Yes	277	37.9
No	453	62.1
**Living arrangements**		
Family	691	94.7
Own	19	2.6
Other‡	20	2.7
**Residential area**		
Cape Town Metro	650	89.0
Rural (outside Cape Town Metro)	80	11.0
**Marital status**		
Single	672	92.1
Married	20	2.7
Divorced	30	4.1
Widowed	6	0.8
Not known	2	0.3
**Primary caregiver**		
Parent or Parents	551	75.5
Spouse	11	1.5
Self	13	1.8
Other§	155	21.2
**Number of children**		
0	552	75.6
1	86	11.8
2	57	7.8
≥ 3	31	4.3
Missing data	4	0.5

† , includes Sotho, isiZulu, Shona, Somali and French.

‡ , includes group home and shelter.

§ , includes brother, sister, aunt, uncle, niece, nephew, cousins, non-marital partner and friends.

The mean age of the cohort was 28 years (range of 18–60; median 27; standard deviation 8.140). Most of the admissions were male (*n* = 600; 82.2%), unmarried (92.1%) and unemployed (92.7%) patients. The most prevalent first language spoken was Afrikaans (75.1%), and most of the patients were educated to at least a secondary level (Grade 7 and above), with only 20.7% having completed their schooling. Only 37.9% were receiving a disability grant. Most of the patients lived in the Cape Town Metro area (89%) and with their families (94.7%). The primary caregivers in most cases were the patients’ parents (75.5%), and most of the patients had no children (75.6%).

Table 2 summarises the clinical characteristics of patients admitted to New Beginnings. The predominant healthcare provider was largely the mental health services at the community health clinic (90.4%), followed by psychiatric hospitals (8.8%) and private psychiatrists (0.8%). Stikland Hospital was the main referring hospital (*n* = 643, 88.1%). Only 9.5% were part of the ACT service. The most common primary psychiatric diagnosis was schizophrenia (53.7%), and a combination of oral and depot treatment (46.8%) was the most common treatment regimen followed.

**TABLE 2:** Clinical characteristics of patients (*N* = 730) admitted to New Beginnings step-up or step-down facility from 01 January 2011 to 31 December 2015.

Variable	*n*	%
**Predominant healthcare provider**		
Community mental health	660	90.4
Psychiatric hospital	64	8.8
Private psychiatrist	6	0.8
**Referring hospital**		
Stikland Psychiatric Hospital	643	88.0
Lentegeur Psychiatric Hospital	80	11.0
Other†	7	1.0
**Assertive community treatment**		
Yes	69	9.5
No	661	90.5
**Primary diagnosis**		
Schizophrenia	392	53.7
Bipolar disorder (I and II)	86	11.8
Schizoaffective disorder	85	11.6
Substance induced psychotic disorder	141	19.3
Other‡	26	3.6
**Treatment regime**		
Single oral	123	16.9
Multiple oral	125	17.2
Depot only	10	1.4
Oral and depot	341	46.7
Clozapine	96	13.2
Clozapine and depot	33	4.5
**Illicit substances**		
None	176	24.1
Cannabis only	174	23.8
Methamphetamine only	74	10.1
Cannabis and methamphetamine	219	30.0
Cannabis, methamphetamine and other§	81	11.1
Other¶	5	0.7
Missing	1	0.1
**Alcohol use disorder**		
Yes	58	7.9
No	672	92.1
**Medical comorbidities**		
None	642	87.9
Single medical condition	77	10.5
Multiple medical conditions	11	1.5
**Psychiatric admissions pre-index NB**		
None	5	0.7
1 admission	258	35.3
2 admissions	159	21.8
Multiple admissions (three or more)	308	42.2
**Total NB admissions**		
1	547	74.9
2	125	17.1
Multiple admissions (three or more)	58	8.0

NB, New Beginnings.

† , includes tygerberg hospital, karl bremer hospital and alexandra hospital.

‡ , includes major depressive disorder, generalised anxiety disorder, post-traumatic stress disorder, intellectual disability and major neurocognitive disorder secondary to traumatic brain injury.

§ , includes cannabis, methamphetamine in combination with methaqualone, cocaine, ecstasy or heroin.

¶ , includes heroin, ecstasy, cocaine and lysergic acid diethylamide (LSD) (in the absence of cannabis and/or methamphetamine).

Illicit substances were used by 75.9% of patients. Thirty per cent of patients used both cannabis and methamphetamine, while 23.9% used only cannabis and 10.2% used only methamphetamine.

Only 11.1% used multiple (three or more) drugs including cannabis, methamphetamine and at least one other substance (commonly methaqualone). An alcohol use disorder was documented in 7.9% (*n* = 58) of admitted patients.

Only 10.5% of patients had one medical comorbidity (including hypertension, diabetes mellitus, dyslipidaemia or primary syphilis) and 1.5% had multiple medical conditions (two or more).

The mean number of psychiatric admissions was 5.07 (range 0–42; SD 4.6), and the mean for total days admitted to hospital was 274.57 days (range 0–3326; SD 335.074).

Most patients had a single admission (74.9%) to New Beginnings, while 17.1% (*n* = 125) had two admissions. The maximum number of admissions was 11 for one patient. While 35.3% of patients had their single index psychiatric hospitalisation prior to admission to New Beginnings, most had three or more previous psychiatric admissions (42.2%).

## Discussion

Our results provide valuable information on the demographic and clinical profile of patients admitted to the facility. The high percentage of male admissions (82.2%) to New Beginnings correlates with studies conducted on psychiatric hospital inpatient acute wards. Franken et al. assessed the profile of adult acute admissions to Lentegeur Psychiatric Hospital and found that most were male patients.^21^ In addition, violence leading up to admission was significantly associated with the male gender as well as substance use. Men are more likely to be violent or disruptive in their community which then leads to inpatient admission,^22^ and subsequently a higher likelihood of having the opportunity of referral to a step-up or step-down facility, and therefore make up the bulk of admissions to these facilities. The lower number of female beds and female admissions to New Beginnings, despite no marked differences in the population rates of severe mental disorders like schizophrenia and bipolar disorder,^23^ might reflect gender bias and would be a valuable area of further research.

A high percentage of patients are living with family members (94.7%), most often parents, as primary caregivers. This likely reflects the need for social support as part of the admission criteria as most patients only have family support. This is necessary as part of the programme is the re-integration with your support system via graded weekend leave for the duration of the admission and family psycho-education. Caregiver burden on those taking care of patients with a serious psychiatric illness negatively impacts on their quality of life.^24^ Families spend large amounts of time taking care of their dependent relatives and, depending on the level of severity, also manage all activities of daily living. Jeyagurunathan et al. provided insight into the psychological status and quality of life of caregivers looking after patients with severe mental illness.^24^ They found that adverse outcomes in terms of caregiver quality of life and mental illness can be expected and that there is a need to include caregivers in psycho-education programmes and social support. This highlights the importance of interventions that involve caregivers in the psychosocial rehabilitation process and provide education and support to optimise reintegration of psychiatric patients back into the community.

The high levels of unemployment found in this sample also highlight the importance of incorporating vocational rehabilitation in a transitional care programme. People with psychiatric disability face unique barriers to employment, including stigma, fluctuating impairment patterns, difficulties with social skills and complex support needs.^25^ In addition to improved employment, people who gain competitive jobs through supported employment experience many benefits related to finances, self-esteem and quality of life.^26^

We noted a high incidence of substance use, specifically methamphetamine and cannabis. There is a growing methamphetamine (Tik) epidemic in the Western Cape. Watt et al. reported on methamphetamine usage in Cape Town and found that methamphetamine use had adverse effects on mental, physical and economic well-being, and limited future opportunities through school dropout and incarceration.^27^ Substance rehabilitation or treatment centres are primarily focused on substance misuse per se, and not aimed at substance misuse in the context of a comorbid mental illness. Psychosocial treatment for methamphetamine use disorders has a strong evidence base and is considered first-line treatment to reduce rates of psychosis by preventing relapse. Thomas et al. recommended the adoption of an integrated, co-ordinated mental health and substance use service that will require acquisition of necessary skills by mental healthcare practitioners.^28^ Drake et al. stipulated that at least three types of integrated interventions for substance use disorder are probably effective for dual diagnosis clients: group counselling, contingency management and residential treatment.^29^ The findings highlight the need for substance interventions that cater specifically for patients with dual diagnoses. Additionally, we recommend that substance use interventions should be included in discharge planning. Training is also necessary to equip staff with the skills to manage dual diagnosis patients.

South Africa has the second highest incidence of alcohol abuse in the world after Ukraine.^30^ These findings do not correlate with this study as low rates of alcohol abuse were reported. One reason for this could be that clinicians may tend to under-document alcohol abuse, focusing rather on illicit substances. Patients and families often may not view alcohol as a substance of abuse, because it is legal and the use thereof is the norm within a large part of our society.^22^ This needs to be addressed and improved on in terms of screening for an alcohol use disorder, because identifying such patients is critical to establish specific dual diagnosis interventions in transitional care facilities.

Schizophrenia was the most common diagnosis among New Beginnings patients. The high rates of schizophrenia diagnosis may possibly be related to high rates of methamphetamine use as methamphetamine-induced psychosis has a similar clinical picture and could confound making the diagnosis.^31^ Research has shown that the intensity, specificity and length of community-based psychosocial rehabilitation services were associated with superior functional outcomes for individuals diagnosed with schizophrenia.^4^ Without community-based rehabilitation, mental healthcare users are likely to find it difficult to reintegrate into the community and have little support for their recovery.^4^

This may put them at risk to default clinic appointments and medication, and inevitably lead to relapses. This is demonstrated by the high number of readmissions in this sample. The average patient admitted to New Beginnings already had three or more psychiatric admissions on average.

Botha et al. stipulated that ACT can successfully be modified in under-resourced settings and sustain reductions in inpatient usage over time.^7^ Such interventions can be successfully incorporated into existing services, tailored according to the needs of the community and resources available, and work in a symbiotic fashion with step-up or step-down facilities such as New Beginnings.

Most patients admitted to New Beginnings were on a treatment regimen consisting of oral treatment as well as a depot antipsychotic. This possibly reflects the significant bed pressure under which acute psychiatric inpatient units operate. Current evidence supports the benefits of using long-term injectable anti-psychotics, specifically in reducing readmissions and improving adherence.^32^ In acute settings where bed utilisation rate is high, patients often require additional oral anti-psychotics, which may contribute to patients being prematurely discharged on combination treatments. This further highlights the need for an intermediary facility that may afford clinicians the time to optimise treatment regimens.

Most patients had at least three psychiatric admissions prior to their first New Beginnings admission. There may be multiple reasons for this. First, as New Beginnings was only established in 2008, some patients had multiple psychiatric hospital admissions prior to the existence of the service. Second, New Beginnings has limited bed capacity (32 male and 8 female) and thus would not allow for all admitted patients to be transferred there. Slightly more than one-third (35.3%) of patients went to New Beginnings after an index psychiatric admission. This is known to be the ideal time to intervene, and ideally, all first episode patients would benefit from psychosocial rehabilitation and gradual return to the community. Multiple admissions lead to longer recovery time, lower social functioning, increased substance abuse, increased crime and larger caregiver burden.^4^ Unfortunately, resources may not allow for all first episode patients to be admitted to New Beginnings. Third, New Beginnings is a voluntary unit and some patients, because of lack of insight, may not be willing to go to New Beginnings for psychosocial rehabilitation. Many inpatient admissions are already longer than 4 weeks, so patients are reluctant to agree to attend a psychosocial rehabilitation programme that will delay their return home for another 8 weeks. Further longitudinal studies are needed to look at whether completing the New Beginnings programme makes a difference towards future psychiatric admissions and the revolving door phenomenon.

A limitation of this retrospective study is that our data depended on information that was documented in the file, which in some instances did not contain all the relevant information needed. This was particularly true for the highest level of education. Furthermore, the retrospective nature of our study limits the inference of causality. Further studies need to build on our findings and determine any relevant significant associations. This may include, for example, exploring: (1) the impact of completing the full programme at New Beginnings on acute readmissions; (2) the association between certain demographic factors, such as gender, highest level of education, relationship status and social support and the likelihood of completion of the programme; and (3) the relationship between substance use and the likelihood of non-completion of the programme. Such studies would be helpful in identifying whether there is a need for more intermediary facilities and whether existing facilities need improvement in, for example, the programme, staff and referral criteria.

## Conclusion

Our study describes the demographic and clinical profile of patients admitted to New Beginnings, a step-up or step-down facility during 01 January 2011 and 31 December 2015. Our study identified male gender, unmarried, unemployed and living with family, for example, parents as prominent demographic features. The high rates of substance use, repeated inpatient admissions and treatment combinations highlight the need for intermediary facilities that afford patients the time to settle fully and provide substance use interventions that are tailored to the needs of patients with dual diagnosis, while giving staff the time to streamline treatment regimens and engage caregivers. We believe that further studies on step-up or step-down facilities for mental illness would help in bridging the growing gap between psychiatric hospital treatment and community care. Further research is needed to determine to what extent the New Beginnings programme improves quality of life and functioning, affects future readmissions or can be shown to be cost-effective. Such research would be helpful to determine how existing step-up or step-down facilities can be made most efficient, and possibly if there is a need for more such facilities in the Western Cape and South Africa.
